# Serum Levels of Toxic AGEs (TAGE) May Be a Promising Novel Biomarker for the Onset/Progression of Lifestyle-Related Diseases

**DOI:** 10.3390/diagnostics6020023

**Published:** 2016-06-07

**Authors:** Masayoshi Takeuchi

**Affiliations:** Department of Advanced Medicine, Medical Research Institute, Kanazawa Medical University, Uchinada-machi, Kahoku, Ishikawa 920-0293, Japan; takeuchi@kanazawa-med.ac.jp; Tel.: +81-76-218-8456; Fax: +81-76-286-3652

**Keywords:** advanced glycation end-products (AGEs), biomarker, toxic AGEs (TAGE), receptor for AGEs (RAGE), cardiovascular disease (CVD), nonalcoholic steatohepatitis (NASH), cancer, Alzheimer’s disease (AD), infertility, lifestyle-related diseases (LSRD)

## Abstract

Advanced glycation end-products (AGEs) generated with aging or in the presence of diabetes mellitus, particularly AGEs derived from the glucose/fructose metabolism intermediate glyceraldehyde (Glycer-AGEs; termed toxic AGEs (TAGE)), were recently shown to be closely involved in the onset/progression of diabetic vascular complications via the receptor for AGEs (RAGE). TAGE also contribute to various diseases, such as cardiovascular disease; nonalcoholic steatohepatitis; cancer; Alzheimer’s disease, and; infertility. This suggests the necessity of minimizing the influence of the TAGE-RAGE axis in order to prevent the onset/progression of lifestyle-related diseases (LSRD) and establish therapeutic strategies. Changes in serum TAGE levels are closely associated with LSRD related to overeating, a lack of exercise, or excessive ingestion of sugars/dietary AGEs. We also showed that serum TAGE levels, but not those of hemoglobin A1c, glucose-derived AGEs, or Nε-(carboxymethyl)lysine, have potential as a biomarker for predicting the progression of atherosclerosis and future cardiovascular events. We herein introduce the usefulness of serum TAGE levels as a biomarker for the prevention/early diagnosis of LSRD and the evaluation of the efficacy of treatments; we discuss whether dietary AGE/sugar intake restrictions reduce the generation/accumulation of TAGE, thereby preventing the onset/progression of LSRD.

## 1. Introduction

Diabetes mellitus (DM) is one of the largest global health emergencies of the 21st century. Increases are reported each year in the number of individuals with this hyperglycemic condition, which may result in life-changing complications. In addition to the 415 million adults (this will increase to 642 million adults by 2040) already estimated to currently have DM (a DM-related death occurs every 6 s), there are 318 million adults with impaired glucose tolerance (IGT), which places them at high risk of developing the disease in the future [1]. Under hyperglycemic conditions, a non-enzymatic glycation reaction (Maillard reaction) between reducing sugars and the amino groups of proteins progresses in an accelerated manner; it begins with the conversion of reversible Schiff base adducts, and then to more stable, covalently-bound Amadori rearrangement products. Over the course of days to weeks, these early glycation products undergo further reactions, such as rearrangements and condensation to become irreversibly cross-linked, fluorescent macroprotein derivatives termed advanced glycation end-products (AGEs) [2,3,4,5]. Continuous hyperglycemia is involved in the pathogenesis of diabetic micro- and macro-vascular complications via various metabolic pathways, and numerous hyperglycemia-induced metabolic and hemodynamic conditions exist, including the increased generation of various types of AGEs [6,7,8,9]. We produced seven specific antibodies for non-Nε-(carboxymethyl)lysine (CML) AGEs that recognized the immunoreactive types of AGEs (glucose-, fructose-, glyceraldehyde-, glycolaldehyde-, methylglyoxal (MGO)-, glyoxal (GO)-, and 3-deoxyglucosone (3-DG)-derived AGEs) [10,11,12,13]. We recently demonstrated that glyceraldehyde-derived AGEs, the predominant components of toxic AGEs (TAGE), play an important role in the pathogenesis of angiopathy in DM patients [7,8,9,14]. Moreover, a growing body of evidence suggests that the interaction between TAGE and the receptor for AGEs (RAGE) alters intracellular signaling, gene expression, and the release of pro-inflammatory molecules, and also elicits reactive oxygen species (ROS) generation in numerous types of cells, all of which may contribute to the pathological changes observed in lifestyle-related diseases (LSRD).

Hence, a focus was placed on TAGE and a specific competitive enzyme-linked immunosorbent assay (ELISA) was developed, and the clinical utility of measuring TAGE as a biomarker for evaluating disease activity in LSRD was examined.

## 2. AGEs

AGEs are generated by the Maillard reaction, a non-enzymatic reaction, between the aldehyde or ketone groups of reducing sugars, such as glucose and fructose, and the N-terminal α-amino group or ε-amino group of the lysine residues of proteins, and contribute to the aging of proteins as well as pathological complications associated with DM [2,3,4,5,6,7,8,9]. In hyperglycemia, this process begins with the conversion of reversible Schiff base adducts to more stable, covalently bound Amadori rearrangement products. Over the course of days to weeks, these Amadori products undergo further rearrangement reactions to generate irreversibly bound moieties known as AGEs. AGEs were originally characterized by their yellow-brown fluorescent color as well as their ability to form cross-links with and between amino groups; however, this term now encompasses a broad range of advanced products of the glycation process, including CML, Nε-(carboxyethyl)lysine (CEL), and pyrraline, which do not display color or fluorescence and are not cross-linked proteins [2,3,4,5]. *In vivo* AGE generation is affected by sugar concentrations, the rate of turnover of the chemically modified target, and the time available. Increases in glucose concentrations were previously considered to have a major influence on the Maillard reaction; however, glucose is one of the least reactive sugars found in biological organisms [2,15]. In addition to extracellular AGE generation, the rapid intracellular generation of AGEs from intracellular precursors such as trioses (*i.e*., glyceraldehyde), dicarbonyl compounds, and fructose has been gaining attention [16,17]. Due to marked variations in the structures of AGEs found *in vivo* and the complex nature of the reactions required for their generation, only some AGEs have had their structures identified to date [18]. The structures of cytotoxic AGEs have not yet been elucidated.

## 3. Alternative Routes for the Generation of Various AGEs *in Vivo*

**Figure 1:**
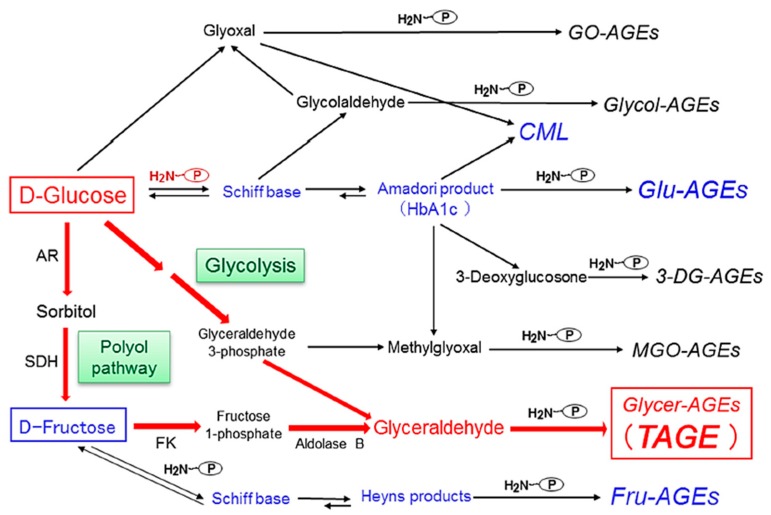
Alternative routes for the generation of advanced glycation end-products *in vivo*: Reducing sugars, such as glucose, fructose, and glyceraldehyde, which are known to react non-enzymatically with the amino groups of proteins to form reversible Schiff bases and Amadori products/Heyns products. These early glycation products undergo further complex reactions, such as rearrangement, dehydration, and condensation, to become irreversibly cross-linked, heterogeneous fluorescent derivatives, termed advanced glycation end-products (AGEs). Glu-AGEs: glucose-derived AGEs; Fru-AGEs: fructose-derived AGEs; Glycer-AGEs: glyceraldehyde-derived AGEs; Glycol-AGEs: glycolaldehyde-derived AGEs; MGO-AGEs: methylglyoxal-derived AGEs; GO-AGEs: glyoxal-derived AGEs; 3-DG-AGEs: 3-deoxyglucosone-derived AGEs; CML: Nε-(carboxymethyl)lysine; P-NH2: free amino residue of a protein; AR: aldose reductase; SDH: sorbitol dehydrogenase; FK: fructokinase; HbA1c: hemoglobin A1c; TAGE: toxic AGEs.

We previously reported the contribution of α-hydroxyaldehydes (glyceraldehyde and glycolaldehyde), dicarbonyl compounds (MGO, GO, and 3-DG), and fructose as well as glucose to the glycation of proteins [10,11,12,13]. Seven immunochemically distinct classes of AGEs (Glu-AGEs, glucose-derived AGEs; Fru-AGEs, fructose-derived AGEs; Glycer-AGEs, glyceraldehyde-derived AGEs; Glycol-AGEs, glycolaldehyde-derived AGEs; MGO-AGEs, MGO-derived AGEs; GO-AGEs, GO-derived AGEs; and 3-DG-AGEs, 3-DG-derived AGEs) have been detected in serum samples collected from diabetic nephropathy on hemodialysis (DN-HD) [10,11,12,13]. Accordingly, the *in vivo* generation of AGEs was suggested to occur via a process involving the Maillard reaction, sugar autoxidation, and sugar metabolic pathways (Figure 1).

## 4. Pathway for the Generation of Glyceraldehyde (GLA) *in Vivo*

**Figure 2:**
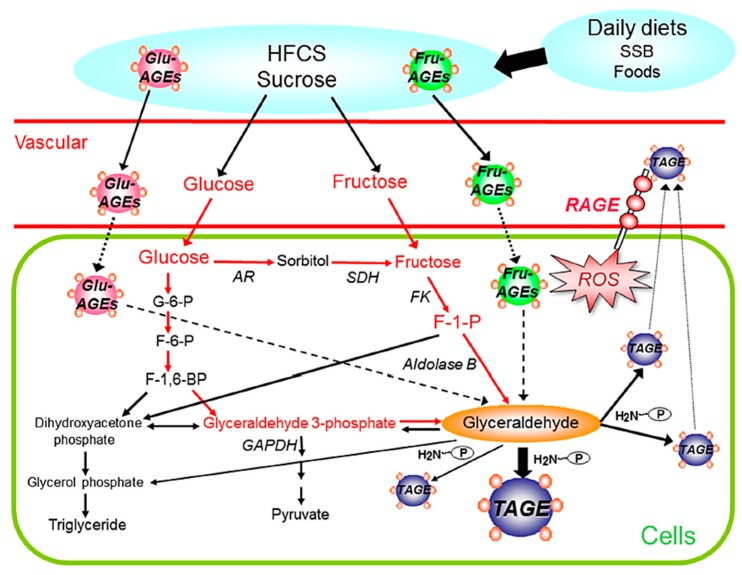
Routes for *in vivo* TAGE generation: The chronic ingestion of excessive amounts of sugar-sweetened beverages (SSB) and commercial food products increases the levels of the sugar metabolite, glyceraldehyde in cells. The glyceraldehyde produced induces the generation of TAGE in intracellular compartments. As a result, TAGE accumulate in cells, cause cell damage, and leak into the blood, and, thus, TAGE levels in circulating fluids may be considered to increase. Furthermore, the chronic ingestion of excessive dietary AGEs (mainly Glu-/Fru-AGEs) increases the enhanced generation/accumulation of TAGE and the expression of RAGE, thereby leading to TAGE-RAGE interactions. Interactions between TAGE and RAGE alter intracellular signaling, gene expression, and the release of pro-inflammatory molecules and also elicit the generation of ROS in numerous types of cells, all of which may contribute to the pathological changes observed in lifestyle-related diseases. TAGE: toxic AGEs; RAGE: receptor for AGEs; ROS: reactive oxygen species; SSB: sugar-sweetened beverages; HFCS: high-fructose corn syrup; AR: aldose reductase; SDH: sorbitol dehydrogenase; FK: fructokinase; GAPDH: glyceraldehyde-3-phosphate dehydrogenase; G-6-P: glucose-6-phosphate; F-6-P: fructose-6-phosphate; F-1,6-DP: fructose-1,6-diphosphate; F-1-P: fructose-1-phosphate; P-NH2: free amino residues of proteins.

Two different pathways are responsible for the *in vivo* generation of GLA, which is the precursor of TAGE: (i) the glycolytic pathway (glycolysis) and (ii) the fructose metabolic pathway (fructolysis) [7,8,9,19]. In pathway (i), the enzyme GLA-3-phosphate (G-3-P) dehydrogenase (GAPDH) generally breaks down the glycolytic intermediate G-3-P. However, reductions in GAPDH activity lead to the intracellular accumulation of G-3-P. Therefore, G-3-P starts to be metabolized via an alternative pathway, causing increases in the concentration of GLA and, as a result, promotes the generation of TAGE. Therefore, a positive feedback mechanism is in operation; namely, the inhibition of GAPDH activity by GLA promotes the generation of TAGE. In pathway (ii), an increase in intracellular glucose concentrations under hyperglycemic conditions stimulates the generation of fructose via the polyol pathway in insulin-independent tissues, such as nerve tissues, the kidneys, the lens of the eyes, red blood cells, and the brain [20,21]. Fructose is a constituent of high-fructose corn syrup (HFCS) and sucrose, and, hence, is commonly included in the human diet [22,23]. Fructokinase phosphorylates fructose to fructose-1-phosphate, which is then broken down into GLA and dihydroxyacetone phosphate by aldolase B [24,25]. The GLA produced induces the generation of TAGE in intracellular compartments. The accumulation of TAGE results in cell damage, TAGE leak into the blood, and, thus, TAGE levels in circulating fluids are considered to increase (Figure 2).

## 5. Methods for the Detection of Serum TAGE Levels

We found that (i) seven distinct classes of AGE structures circulate in the blood of individuals with DN-HD [10,11,12,13]; (ii) the neurotoxic effects of the serum fraction from DN-HD patients containing various AGE structures are completely neutralized by the addition of antibodies raised against TAGE [26]; and (iii) TAGE mimic the deleterious effects of AGE-rich serum purified from DN-HD on endothelial cells (EC) [27]. Furthermore, due to their stronger binding affinity to the receptor for RAGE [28,29], TAGE are considered to be more cytotoxic than other AGEs. Hence, we developed a specific competitive ELISA for TAGE.

### 5.1. Preparation of an Anti-TAGE-Specific Antibody

An immunopurified anti-TAGE antibody was prepared as described in our previous studies [11]. Briefly, 4 mg of TAGE-rabbit serum albumin (RSA) was emulsified in 50% Freund’s complete adjuvant and then injected intradermally into Japanese white rabbits. This procedure was repeated at weekly intervals for 6 weeks. After a 2-week break, the rabbits were given a booster injection of 4 mg of the TAGE-RSA. The animals were bled on the 10th day after the last injection, and their sera were obtained for further affinity purification. A CNBr-activated Sepharose 4B gel was coupled to TAGE-bovine serum albumin (BSA) as described previously [11]. The anti-TAGE antiserum, which contained anti-TAGE and anti-CML antibodies, was applied to a column (2.5 × 5.5 cm) containing Sepharose 4B coupled to TAGE-BSA. After washing with phosphate-buffered saline (PBS), the adsorbed fractions were eluted with 20 mM sodium phosphate buffer containing 1 M potassium thiocyanate (pH 7.4). The eluted fractions were pooled, concentrated using Centriprep-10, and passed through a PD-10 column equilibrated with PBS. The eluted fraction was then loaded onto a column (1.5 × 5.5 cm) containing Sepharose 4B coupled with CML-BSA, which was washed with PBS to obtain the unadsorbed fraction (anti-TAGE antibody). The anti-TAGE antibody was pooled, concentrated with Centriprep-10, and passed through a PD-10 column equilibrated with PBS, before being used in an ELISA [11].

The immunopurified anti-TAGE antibody did not recognize well-characterized AGE structures, such as CML, CEL, pyrraline, pentosidine, argpyrimidine, imidazolone, GO-lysine dimers, MGO-lysine dimers, and GLA-derived pyridinium. Furthermore, it did not recognize AGEs with unknown structures, such as Glu-AGEs, Fru-AGEs, Glycol-AGEs, GO-AGEs, MGO-AGEs, and 3-DG-AGEs [11,27,30]. Instead, the anti-TAGE antibody specifically recognized unique unknown Glycer-AGE structures. The anti-TAGE antibody was able to detect high- and low-molecular-weight TAGE with unique unknown structures in human/animal sera [11,27,30].

### 5.2. Competitive ELISA for Serum TAGE Levels

Serum TAGE levels were measured with a competitive ELISA using an immunopurified anti-TAGE antibody as described previously [11]. Briefly, 96-well (flat-bottomed without a lid, high binding) enzyme/radio immunoassay plates were coated with 1 μg/mL TAGE-BSA standard solution in each well and incubated at 4 °C overnight. The wells were washed three times with 0.3 mL of the washing solution (PBS containing 0.05% Tween-20). Wells were then blocked by being incubated for 1 h with 0.2 mL of a solution of PBS containing 1% BSA. After washing with the washing solution, test samples (50 μL) were added to each well as a competitor for 50 μL of the immunopurified anti-TAGE antibody (1:1000), followed by an incubation for 2 h at 30 °C with gentle shaking on a horizontal rotary shaker. Wells were then washed with washing solution and developed with alkaline phosphatase-linked anti-rabbit IgG utilizing p-nitrophenyl phosphate as the colorimetric substrate. The TAGE concentrations of each sample were read from the calibration curve for the TAGE-BSA standard and were expressed as TAGE units (U) per mL, where 1 U corresponded to 1 μg of the TAGE-BSA standard [30,31]. The sensitivity and intra- and inter-assay coefficients of variation were 0.01 U/mL, 6.2%, and 8.8%, respectively [30,31].

## 6. Clinical Relevance of Serum TAGE Levels and LSRD

We recently demonstrated that TAGE play an important role in the pathogenesis of angiopathy in DM patients [7,8,9,14]. Moreover, a growing body of evidence suggests that the interaction between TAGE and RAGE alters intracellular signaling, gene expression, and the release of pro-inflammatory molecules, and also elicits ROS generation in numerous types of cells, all of which may contribute to the pathological changes observed in diabetic vascular complications, cardiovascular disease (CVD), nonalcoholic steatohepatitis (NASH), cancer, Alzheimer’s disease (AD), and infertility [7,8,9,14,30,31,32,33,34,35,36,37,38,39,40,41,42,43,44]. Therefore, the inhibited formation of TAGE, blockade of TAGE-RAGE interactions, and suppression of RAGE expression or its downstream pathways are promising targets for therapeutic interventions against LSRD.

### 6.1. Non-DM/DM

There is a growing body of evidence to suggest that continuous hyperglycemia under diabetic conditions enhances the generation of AGEs, which are senescent macroprotein derivatives, through non-enzymatic glycation. We recently demonstrated that TAGE play an important role in the pathogenesis of angiopathy in DM patients [7,8,9]. Furthermore, interactions between TAGE and RAGE have been shown to alter intracellular signaling, gene expression, and the release of pro-inflammatory molecules and also elicit the generation of ROS in numerous types of cells (such as EC, pericytes, mesangial cells, tubular cells, and podocytes), all of which may contribute to the pathological changes associated with diabetic vascular complications [7,8,9].

The findings of our recent studies revealed that serum TAGE levels, but not those of hemoglobin A1c (HbA1c), Glu-AGEs or CML were (i) associated with thrombogenic markers (*i.e*., plasminogen activator inhibitor-1 and fibrinogen) and low-density lipoprotein cholesterol levels in a general population [45,46,47]; (ii) correlated with serum pigment epithelium-derived factor (PEDF) and dipeptidyl peptidase-4 (DPP-4) levels, markers of insulin resistance (IR), in a general population [48,49]; (iii) associated with visceral and subcutaneous adipose tissue inflammation and decreased adiponectin levels in outpatients [50,51]; (iv) correlated with vascular inflammation and endothelial dysfunction in high-risk patients [30,52]; (v) elevated under chronic kidney disease (CKD) and/or DM conditions and correlated with inflammatory biomarkers such as monocyte chemoattractant protein-1 (MCP-1), the soluble form of vascular cell adhesion molecule-1 (sVCAM-1) and asymmetric dimethylarginine (ADMA) [53,54,55,56,57]; (vi) correlated with a soluble form of RAGE (sRAGE) that may reflect tissue RAGE expression in non-DM and DM subjects [53,54,58,59], thereby suggesting their utility as a marker for the activation of the TAGE-RAGE axis. Moreover, statin (a hydroxymethyl-glutaryl (HMG)-CoA reductase inhibitor), α-glucosidase, and the DPP-4 inhibitor, sulfonyl urea, have been shown to significantly decrease serum TAGE levels, which are associated with reduced biomarker levels for organ damage in DM or CKD subjects [60,61,62,63,64,65,66]. These findings indicate that serum TAGE levels, but not those of HbA1c, CML, or Glu-AGEs, have potential as a biomarker to predict the onset/progression of LSRD.

### 6.2. CVD

There is a growing body of evidence, ranging from *in vitro* experiments to pathological analyses and epidemiological studies, to suggest that atherosclerosis is intrinsically an inflammatory disease [67,68]. A recent study found that an acarbose treatment reduced the rate at which the intimal media of the carotid arteries thickened in patients with DM or IGT, and led to a lower incidence of CVD [69], indicating that acarbose ameliorates postprandial hyperglycemia, and, hence, inhibits the onset/progression of CVD. We reported that the activation of the TAGE-RAGE axis resulted in the generation of intracellular ROS and the subsequent activation of nuclear factor-κB in vascular wall cells, which may promote the expression of various atherosclerosis- and inflammation-related genes, thereby contributing to the onset/progression of CVD in DM [6,9,70].

In our previous study, serum TAGE levels, but not those of HbA1c, were found to be a marker of cumulative postprandial hyperglycemia in DM rats treated with nateglinide [71]. We demonstrated that serum TAGE, but not HbA1c levels, in DM patients decreased significantly after a 12-week acarbose treatment [64]. These findings indicate that HbA1c levels do not accurately reflect the ameliorative effects of acarbose on postprandial hyperglycemia. We suggest that serum TAGE levels may be a useful biomarker for assessing cumulative postprandial hyperglycemia in DM patients.

Endothelial progenitor cells (EPC) contribute to maintaining the structure and function of the endothelium, and, hence, facilitate angiogenesis and vascular repair. In addition, the number of circulating EPC and their activity levels were found to be inversely correlated with atherosclerotic risk factors. Thus, the number and activity levels of EPC may be useful biomarkers for predicting cardiovascular events. In recent studies, we found that: (i) TAGE levels, but not those of HbA1c or CML were independently associated with vascular inflammation, as evaluated by [^18^F] fluorodeoxyglucose-positron emission tomography (FDG-PET) in outpatients [30]; (ii) TAGE levels were one of the independent correlates of the decreased cell number and impaired migratory activity of circulating EPC in apparently healthy subjects [44], thereby suggesting the involvement of TAGE in impaired EC repair; and (iii) high baseline TAGE levels were associated with plaque progression in an assessment of pitavastatin and atorvastatin in an acute coronary syndrome trial (The JAPAN-ACS Sub-study) in Japan [72]. These findings indicated that serum TAGE levels, but not those of HbA1c or CML, have potential as a biomarker for predicting the progression of atherosclerosis and future cardiovascular events.

### 6.3. NASH

Nonalcoholic fatty liver disease (NAFLD) ranges from simple steatosis to NASH, leads to fibrosis and potentially to cirrhosis, liver failure, and hepatocellular carcinoma (HCC), and is one of the most common causes of liver disease worldwide. NAFLD has also been implicated in other medical conditions such as IR, obesity, metabolic syndrome (MetS), hyperlipemia, hypertension, CVD, and DM.

We previously demonstrated that TAGE induced fibrogenesis- and inflammation-related gene and protein expression, such as that of transforming growth factor-β1 (TGF-β1), collagen type Iα2, and MCP-1, in cultured human hepatic stellate LI90 cells via the NADPH oxidase-derived generation of ROS [33]. Regarding the effects of TAGE on hepatocytes, we recently reported that TAGE-RAGE interactions stimulated hepatic C-reactive protein (CRP) in human hepatoma Hep3B cells via the activation of Rac-1 [73]. We demonstrated that GLA, which is a precursor of TAGE, induced concentration- and time-dependent cell death and increased the intracellular concentration of TAGE in Hep3B cells [74]. We also showed that a TAGE-modified protein of 70 kDa, which we identified as heat shock cognate 70, was detected the earliest and in the greatest abundance in GLA-treated Hep3B cells. We found that intracellular TAGE increased the mRNA expression of the acute phase reactant CRP [74]. These findings prompted us to speculate that extracellular and intracellular TAGE may play roles in the pathogenesis of NAFLD/NASH. The excessive intake of fructose contributes to the onset of NAFLD and to the progression of the disease to NASH. Fructose is metabolized to GLA. We showed that intracellular TAGE was formed in the presence of fructose. We demonstrated that TAGE ameliorated IR in mice fed a high-fat, high-fructose diet [75]. These findings suggest that hepatic TAGE are useful markers for the diagnosis and therapeutic evaluation of IR, and may play a pathological role in the onset of IR. Additionally, heterogeneous nuclear ribonucleoprotein M (hnRNPM) was identified as one of the target proteins for TAGE [76]. These findings suggest that TAGE-modified hnRNPM, resulting from the exposure of cells to fructose, alters gene expression and causes adverse effects in Hep3B cells.

The findings of our recent studies revealed that (i) the formation of TAGE was enhanced during NASH, and serum and hepatic TAGE levels, but not those of Glu-AGEs or CML, were significantly higher in patients with NASH than in healthy controls or patients with simple steatosis [50]; (ii) atorvastatin reduced serum TAGE levels in NASH patients with dyslipidemia [77]. These findings suggest that TAGE play critical roles in the pathogenesis of NASH and may serve as potential targets for therapeutic interventions [34,40,78,79].

### 6.4. Cancer

TAGE are known to be cytotoxic *in vitro* and findings from animal studies suggest their involvement in the pathogenesis of IR and DM, as well as their complications. Human studies have suggested the involvement of TAGE in the onset of vascular inflammation [30], NASH [50], AD [80], and some rare disorders [81,82]. In addition to their potential direct effects, some evidence indicates that TAGE interact strongly with RAGE to cause inflammatory and oxidative responses, which have been implicated in the onset of various cancers [83,84,85].

We previously reported that TAGE stimulated the growth and migration of human melanoma G361 cells [35]. Moreover, tumor formation by melanoma cell xenografts in athymic mice was prevented by neutralizing antibodies raised against RAGE. In tumor-bearing mice, survival rates were prolonged, and a treatment with an anti-RAGE antibody inhibited spontaneous pulmonary metastases of melanoma. In addition, TAGE were detected in the beds of human melanoma tumors, whereas their levels were negligible in normal skin. These findings demonstrate that TAGE may play roles in the growth and invasion of melanoma by interacting with RAGE. We recently found that a high-affinity DNA aptamer directed against TAGE (TAGE-aptamer) blocked the progression of diabetic nephropathy in an animal model of obesity and DM [86]. Furthermore, TAGE-aptamer significantly inhibited the *in vivo* tumor growth of G361 cells [87]. Immunohistochemical and Western blot analyses of G361 cells revealed that the TAGE-aptamer decreased the expression levels of RAGE and vascular endothelial growth factor (VEGF) [87]. We recently found that TAGE increased viable cell numbers and up-regulated the mRNA levels of RAGE and VEGF in human breast cancer MCF-7 cells [39]. A neutralizing anti-RAGE antibody blocked TAGE-induced increases in viable cell numbers, whereas metformin completely suppressed TAGE-induced proliferation as well as the up-regulation of RAGE and VEGF mRNA levels in MCF-7 cells. We also found that TAGE enhanced the migration capacity of human lung adenocarcinoma A549 cells by activating Rac1 via ROS generation and increased their invasion capacity [36]. 

We very recently reported that circulating TAGE levels were significantly higher in non-B or non-C (NBNC) HCC patients than in NASH subjects without HCC or control subjects [88]. In a multiple stepwise regression analysis, age, γ-glutamyl transpeptidase, and high-density lipoprotein cholesterol (inversely) remained significant and were independently related to TAGE levels. These findings suggest that TAGE are involved in the pathogenesis of NBNC-HCC, and, thus, have potential as biomarkers with the ability to discriminate NBNC-HCC from NASH. Very recently, we also found that circulating TAGE levels show a strong positive association with the increased risk of rectal cancer, but no association with the risk of colon cancer, in the nested case-control European Prospective Investigation into Cancer and Nutrition (EPIC) cohort study [89]. In this prospective study in European populations, circulating TAGE were not associated with an overall risk of colorectal cancer. Further research is needed in order to investigate the roles of TAGE in the onset of colorectal cancer.

### 6.5. AD and Schizophrenia

AD is the most common cause of dementia in developed countries. It is characterized pathologically by the presence of senile plaques (SPs) and neurofibrillary tangles (NFTs), the major constituents of which are amyloid β (Aβ) protein and tau protein, respectively [41,90]. AGEs, senescent macroprotein derivatives, formed at an accelerated rate under normal aging, may be identified immunohistochemically in SPs and NFTs in AD patients. Furthermore, recent clinical evidence has suggested that DM is one of the risk factors for the onset/progression of AD [91,92]. In our previous studies, we confirmed that TAGE were strongly neurotoxic in a neuronal culture system [26,41]. The neurotoxicities of TAGE were stronger than those of Glu-AGEs and CML, two extensively examined AGE species. Moreover, the neurotoxic effects of serum AGEs from DN-HD patients were completely attenuated by the addition of an anti-TAGE antibody, but not the antibodies of other AGEs [26,41]. In AD brains, TAGE were mainly detected in the cytosol of neurons in the hippocampus and parahippocampal gyrus, but not in SPs or astrocytes [80]. These findings suggest that the production of TAGE during sugar metabolism may not only be cytotoxic to hepatocytes, but also to neuronal cells and possibly many other cells, thereby inducing cellular and organ impairments. 

Evidence obtained over the past 20 years has indicated that Aβ42 levels in the cerebrospinal fluid (CSF) of AD patients are significantly lower than those in age-matched healthy elderly controls, whereas total tau and p-tauT181 levels are significantly higher [93]. Furthermore, the levels of other AD biomarkers, such as VEGF [94] and TGF-β1 [95], were found to be higher in the CSF of AD patients. We recently demonstrated that intracellular TAGE generation decreased Aβ42 levels and increased total tau and p-tauT181 levels in culture media and also increased the intracellular levels of total tau, p-tauT181, VEGF, and TGF-β in human neuroblastoma SH-SY5Y cells [96]. Although the exact mechanisms underlying the target of TAGE and its downstream signaling pathway currently remain unclear, the measurement of TAGE levels in the CSF and/or serum may be a useful biomarker for the early detection of AD [97,98].

We recently demonstrated that TAGE and sRAGE levels were significantly higher and lower, respectively, in patients with acute schizophrenia, and remained stable over the clinical course [99]. The ratio of TAGE/sRAGE was also significantly higher in patients with schizophrenia than in healthy control subjects. Hence, TAGE and TAGE/sRAGE ratios remain useful diagnostic markers of schizophrenia. However, schizophrenia has been regarded as a functional disease in neuropathological studies of patients with schizophrenia who lack evidence of prominent neurodegeneration, indicating the differing roles of increased TAGE levels in schizophrenia and neurodegenerative diseases.

### 6.6. Infertility

AGEs accumulate with aging and DM and play pivotal roles in their pathogenesis. Aging and polycystic ovary syndrome, a similar disease to DM, are the most common causes of infertility. TAGE have been implicated in LSRD, such as IR and postprandial hyperglycemia. Infertile female patients frequently develop IR and IGT. The relationships between serum TAGE levels and the numbers of oocytes collected or ongoing pregnancy rates have been examined, and the findings obtained demonstrated that both factors decreased with age and that ongoing pregnancy rates were lower in the group with higher serum TAGE levels than in those with lower levels, even at younger ages [31]. In addition, serum TAGE levels correlated with follicle development, fertilization, embryo development, and pregnancy in assisted reproductive technologies (ART), suggesting that TAGE accumulation is a novel and useful indicator of poor responders that is independent of age and day-3 follicle-stimulating hormone [31]. Non-pregnant poor responders were treated with the DPP-4 inhibitor, sitagliptin, and underwent ART again; ovarian dysfunction improved and ongoing pregnancy rates significantly increased in the group with serum TAGE levels that were reduced by the sitagliptin treatment. Furthermore, sitagliptin significantly enhanced follicular and embryonic development. Clinical and ongoing pregnancy rates were significantly higher in patients treated with sitagliptin (20% and 14%, respectively) than in controls (2.3% and 0%) (unpublished data).

Infertility treatments using TAGE as an indicator are useful for the early diagnosis of ovarian dysfunction. Improvements in the accumulation of TAGE may become a novel therapeutic strategy for a poor ovarian response. Thus, serum TAGE levels may serve as a biomarker for assessing the efficacy of the prevention, early diagnosis, and treatment of ovarian dysfunction.

The clinical relevance of serum TAGE levels was summarized in Table 1.

## 7. Prevention of the Generation/Accumulation of TAGE in LSRD 

Previous studies reported that the chronic ingestion of excessive amounts of sugar/HFCS-containing foods/beverages not only caused obesity and MetS, but was also involved in the onset/progression of CDV, NASH, and AD; however, the underlying mechanisms currently remain unknown [100,101]. We previously revealed that TAGE strongly correlated with LSRD. The chronic ingestion of excessive amounts of sugar-sweetened beverages (SSB), which contain HFCS, sucrose, and dietary AGEs, increased the levels of the sugar metabolite, GLA in the liver. GLA is known to react non-enzymatically with the amino groups of proteins to generate TAGE, enhance the generation/accumulation of TAGE, up-regulate RAGE mRNA levels, and increase serum TAGE levels, leading to TAGE-RAGE interactions. We also demonstrated that increases in hepatic RAGE expression and the enhanced generation/accumulation of TAGE after the oral consumption of Glu-AGE-rich beverages by normal rats play important roles in the pathogenesis of vascular damage [102]. SSB, which contain large amounts of sugar [103] and/or dietary AGEs [104], need to be taken into consideration for disease prevention, particularly in individuals at high risk of developing LSRD.

### 7.1. Dietary AGEs

#### 7.1.1. TAGE Generation/Accumulation

Two major sources of AGEs, exogenous and endogenous AGEs, have been identified in humans [105,106,107,108]. We recently indicated that serum levels of TAGE, but not those of HbA1c, Glu-AGEs, or CML, have potential as a biomarker to predict the progression of LSRD [30,44,64,72,109]. We also reported an increase in the expression of hepatic RAGE and the enhanced the generation/accumulation of TAGE in normal rats administered Glu-AGE-rich beverages that did not contain TAGE [102]. These findings indicate that Glu-AGEs, which are normally contained in beverages/foods [104], and are taken orally into the body, enhance the generation/accumulation of TAGE and increase serum TAGE levels, leading to TAGE-RAGE interactions [102,110].

#### 7.1.2. AGE Content in Beverages and Foods

**Figure 3:**
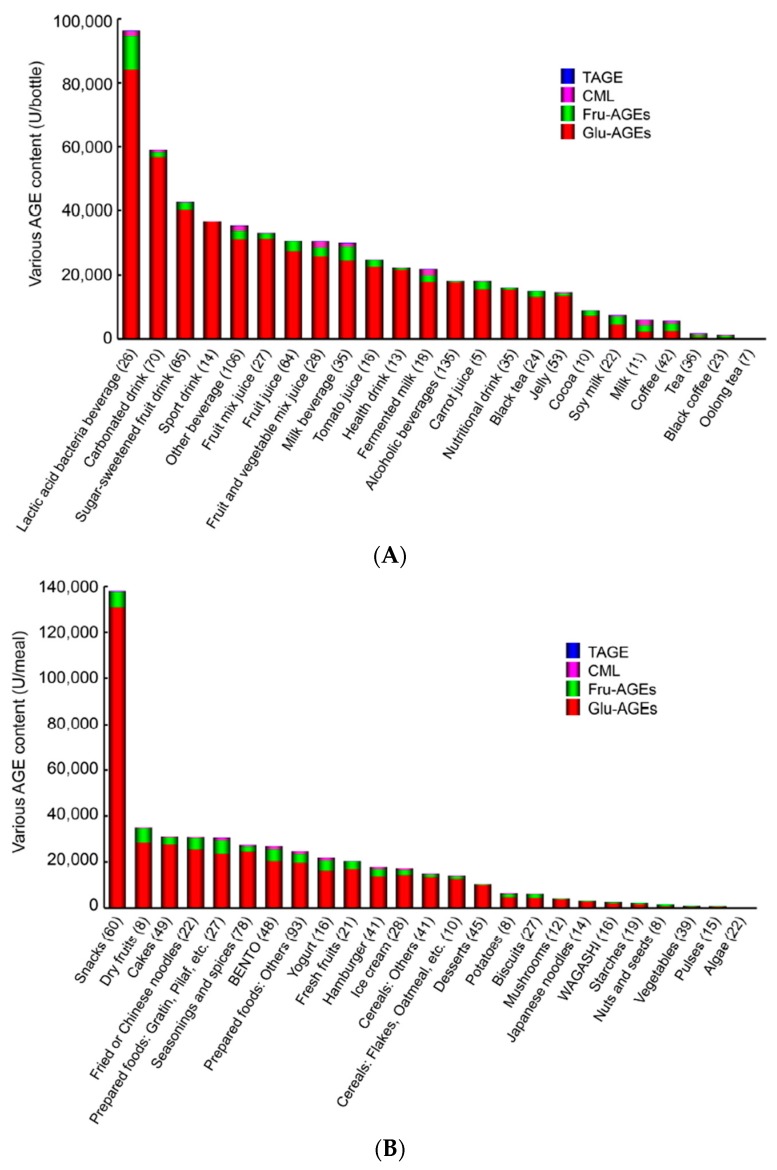
(**A**) Average total AGE contents in commonly consumed beverages in Japan (modified from [104]). Beverages were classified according to the Japanese Agricultural Standard (JAS). Average total AGE contents in commonly consumed beverages in Japan (modified from [104]). Beverages were classified according to the Japanese Agricultural Standard (JAS); (**B**) Average total AGE contents in commonly consumed foods in Japan (modified from [104]). Foods were classified according to the Standard Tables of Food Composition 2009.

Dietary consumption has recently been identified as a major environmental source of pro-inflammatory AGEs in humans. It is currently being disputed whether dietary AGEs represent a risk to human health. CML, a representative AGE compound found in food [111,112], has been suggested to make a significant contribution to circulating CML levels. However, recent studies have found that the dietary intake of AGEs is not associated with plasma CML concentrations [113,114]. We confirmed that serum TAGE levels, but not those of HbA1c, Glu-AGEs, or CML, have potential as a biomarker for predicting the progression of LSRD [30,44,64,72,109]. Therefore, we assessed the concentrations of various AGEs in 1650 beverages and foods commonly consumed in Japan. The concentrations of four kinds of AGEs (Glu-AGEs, Fru-AGEs, CML, and TAGE), which have been detected in the sera of non-DM and DM subjects [30,44,64,72,109,110], were measured with competitive ELISA involving immunopurified specific antibodies [10,11,12,13]. The findings of the latter assays indicated that Glu-AGEs and Fru-AGEs (particularly Glu-AGEs), but not CML or TAGE, were present at appreciable levels in beverages and foods commonly consumed in Japan. Glu-AGEs, Fru-AGEs, CML, and TAGE exhibited concentrations of ≥85%, 2%–12%, <3%, and trace amounts in the beverages examined and ≥82%, 5%–15%, <3%, and trace amounts in the tested foods, respectively (Figure 3A,B) [104].

In humans, it has been demonstrated that roughly 10% of AGEs (estimated CML) in beverages and foods are taken into the body. Of these, ~33% are excreted in urine within 48 h of their consumption, while ~67% accumulate within the body [115]. We examined serum Glu-AGE levels in healthy subjects and Japanese diabetic nephropathy patients and found that they were 10–20 U/mL and 30–50 U/mL, respectively. The consumption of 50,000 U of dietary Glu-AGEs resulted in a blood Glu-AGE level of 1.0 U/mL (50,000 (U) × 0.1 (the proportion that is absorbed) × 1/5000 (mL of blood)). The dietary intake of food products containing <20,000 U Glu-AGEs has been shown to have negligible effects on the body. On the other hand, care is needed when mixing Glu-AGE-containing products or consuming large amounts of Glu-AGE-containing beverages or foods because it may result in elevated concentrations of Glu-AGEs and sugar in the blood and promote the hepatic accumulation of Glu-AGEs [102,110].

#### 7.1.3. Restricting the Consumption of Dietary AGEs

Previous studies have indicated that it is important to consider the amounts of Glu-AGEs present in foods as a step to prevent liver disease, particularly in individuals at risk of DM, CVD, NASH, or chronic renal failure (CRF). Kremezin, an oral adsorbent that slows the onset of CRF by promoting the removal of uremic toxins, was found to reduce serum Glu-AGE and TAGE levels in non-DM CRF patients [110]. Furthermore, in an examination of the expression profiles of EC extracted from the serum samples of the latter patients, the mRNA expression levels of MCP-1, VCAM-1, and RAGE were found to be significantly lower in cells acquired after a Kremezin treatment than in EC obtained prior to the treatment [110]. These findings indicate that the pathogenesis of vascular damage is influenced by dietary Glu-AGEs under TAGE-RAGE-related conditions and that reducing the amount of dietary Glu-AGEs taken into the body represents a useful strategy against LSRD. Further clinical studies may provide novel insights into whether restricting the consumption of dietary AGEs is beneficial for preventing or slowing the progression of LSRD. These findings suggest that the renoprotective and anti-atherosclerotic properties of Kremezin are due to, at least in part, its AGE-lowering ability via the inhibited absorption of AGEs. Therefore, the adsorption of dietary AGEs or their precursors in the intestines by Kremezin has potential as a promising therapeutic strategy for the treatment of AGE-related disorders including progressive renal disease and accelerated atherosclerosis.

There is accumulating evidence to show the pathological role of dietary AGEs in various cardiometabolic disorders and aging. However, additional long-term high-quality randomized studies are needed in order to clarify whether dietary AGE restrictions alleviate the pro-inflammatory and pro-oxidative milieu and insulin sensitivity and protect against cardiovascular and renal damage in healthy individuals and high-risk patients for CVD [106,116].

### 7.2. Sugars (HFCS/Sucrose)

The sugar additives (typically HFCS or sucrose) found in many SSB and commercial products are widely viewed as the main source of the increased amounts of fructose consumed in developed countries. Dyslipidemia, obesity, and IR are all strongly associated with greater fructose consumption, and evidence to show that fructose is involved in the onset/progression of NAFLD is increasing human studies have linked fructose consumption to hepatic fat accumulation, fibrosis, and inflammation. In adolescents, increased fructose consumption is linked with various CVD risk factors. However, visceral obesity may be responsible for these associations. In the United States, fructose consumption is considered to be associated with the recent increases in the prevalence rates of obesity, fatty liver, and DM. The liver is extremely sensitive to variations in dietary content and plays the primary role in the metabolism of simple sugars, such as fructose and glucose [117,118].

#### 7.2.1. TAGE Generation/Accumulation

A growing body of epidemiological and mechanistic evidence argues that excessive sugar consumption affects human health beyond the simple addition of calories [119]. Sugar has been implicated in the onset of all diseases associated with MetS [120,121], including hypertension, CVD, NAFLD/NASH, DM, and the aging process, which is promoted by damage to proteins due to the non-enzymatic binding of sugars (so-called glycation) [2,3,4,5]. We recently reported that the glucose- and fructose-induced generation of GLA caused TAGE, which may be used as biomarkers to predict LSRD [30,44,64,72,109].

#### 7.2.2. Sugar Content in Beverages

The increased consumption of SSB has been observed not only in Western, but also in Asian countries [122], and has been extensively associated with an increased risk of DM and also with weight gain, obesity, MetS, hypertriglyceridemia, coronary heart disease, and hypertension [123,124,125,126,127,128,129]. In the setting of a pandemic of obesity and DM, the American Heart Association (AHA) has recently released scientific recommendations to reduce added-sugar intake to no more than 100 (for women)–150 (for men) kcal (25–37.5 g sugar)/day for most Americans [130]. A new World Health Organization (WHO) guideline in 2015 has recommended that adults and children reduce their daily intake of added sugars to less than 10% of their total energy intake (50 g sugar for a 2000 kcal/day diet). An additional reduction to less than 5% of the total energy intake or roughly 25 g sugar/day may provide additional health benefits [131]. These limits are markedly exceeded by today’s society [132].

**Figure 4:**
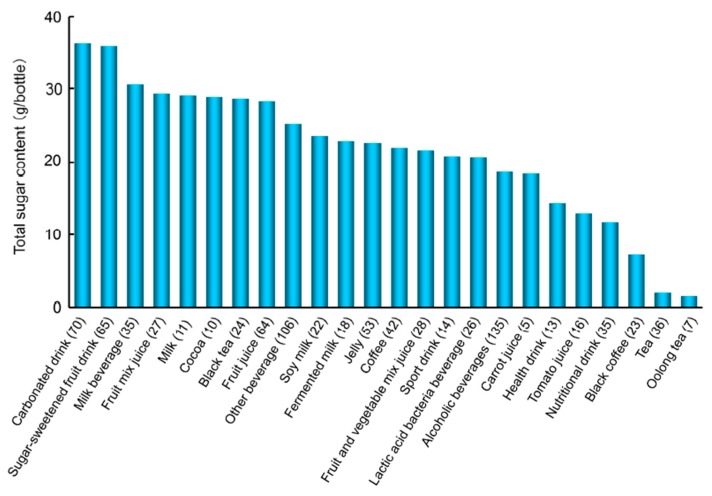
Average total sugar contents in commonly consumed beverages in Japan (modified from [103]). Beverages were classified according to the JAS.

We recently reported the amounts of total sugar and free glucose and calculated fructose plus sucrose in a typical beverage in Japan. Approximately 40% of the beverages tested contained 25 g or more sugar per bottle based on standard serving sizes (Figure 4) [103]. This is the upper limit of daily sugar intake recommended in the guidelines by the AHA [130] and WHO [131] to prevent health conditions in women and adults/children, respectively. A 500-mL bottle of a carbonated drink (Coke, Sprite, or Fanta) contains approximately 50–60 g of added sugars; therefore, the consumption of one bottle equals the recommended amount of added sugars for one day. The amount of glucose was ≥10 g/bottle (the amount of glucose that is prescribed during hypoglycemia to DM patients in Japan) in *ca.* 32% of the beverages examined [103].

#### 7.2.3. Restricting the Consumption of SSB

We evaluated the amounts of AGEs and sugars in beverages and foods, which cause the generation of TAGE in the body. We evaluated the amounts of various AGEs in 885 kinds of beverages and 767 kinds of food, and found that the amounts of AGEs derived from Glu-AGEs/Fru-AGEs (particularly Glu-AGEs) more accurately reflected the amounts of AGEs in beverages and foods than those of CML [104]. Our evaluation of the sugar content of 885 kinds of beverages showed that approximately 40% contained more than the standard intake of sugar (25 g/day) recommended by the AHA and WHO [103]. The habitual intake of more than a small amount of SSB (360 mL/week) in the long-term been reported to increase the risk of the onset of DM [133].

Imamura *et al.* prospectively examined the relationship between the consumption of SSB and risk of DM from 17 cohorts (38,253 cases/10,126,754 person years) [134]. They repeated a meta-analysis to estimate the relative risk for 250 mL/day. The higher consumption of SSB was associated with a greater incidence of DM, by 18% per one serving/day and 13% before and after adjustments for adiposity. The habitual consumption of SSB was associated with a greater incidence of DM, independent of adiposity. These findings suggest that the continued intake of negligible amounts of SSB increases the risk of DM. The consumption of SSB has been directly and indirectly linked to an increased risk of DM. Extensive and lasting changes in public policies are needed in order to curb the worldwide obesity and DM epidemics, and limiting the consumption of SSB may be an important strategy to achieve this.

The findings of our studies suggest that sugars (glucose, fructose, and sucrose) are present at appreciable levels in common beverages [103], and exogenous dietary Glu-AGEs [104] may contribute to the accumulation of TAGE in the body. The contents of HFCS/sucrose and dietary AGEs in beverages/foods need to be taken into consideration for disease prevention, particularly in individuals at high risk of developing LSRD. Additional clinical investigations may provide us with more information as to whether the restriction of dietary sugars and Glu-AGEs is beneficial for preventing the progression of LSRD and represents a novel therapeutic target to prevent these diseases.

These findings are promising in that the concept of the restricted intake of AGEs and sugars in beverages and foods may be a new strategy when considering the suppressed generation/accumulation of TAGE and prevention of LSRD.

## 8. Conclusions and Perspectives

As described above, changes in serum TAGE levels are closely associated with MetS and IR, postprandial hyperglycemia, dyslipidemia, and hypertension, which are related to overeating, the lack of exercise, or excessive ingestion of sugars (HFCS and sucrose)/dietary AGEs (Figure 2). We proposed that the “TAGE theory” strongly correlates with the onset/progression of LSRD, and also suggested the potential of serum TAGE levels as a biomarker for the prevention/early diagnosis of LSRD and evaluation of the efficacy of treatments. Our series of studies has indicated that serum TAGE levels have potential as a novel biomarker for vascular injury and may predict future cardiovascular events in non-DM/DM. The measurement of TAGE using specific competitive ELISA may identify high-risk patients and provide us with valuable information for treatment decision-making in the future. The structure of the epitope recognized by the anti-TAGE antibody was not determined; however, we established that the anti-TAGE antibody differed from those of well-defined AGEs, as well as those of AGEs derived from carbonyl or sugar molecules with unknown structures. Accordingly, it is possible that TAGE have unique structures; however, studies involving spectroscopic and biochemical analyses are required in order to confirm this.

The characteristics of modern dietary habits (excessive intake of glucose, fructose, and AGE-rich foods and beverages) promote the generation and accumulation of TAGE in the body, and are strongly involved in the onset and progression of LSRD. Thus, our findings provide a new concept of preventative measures against LSRD, including aging.

## Figures and Tables

**Table 1:** Clinical relevance of serum TAGE levels.

Subjects	Correlation Factor	Therapeutic Agents	References
Apparently healthy	EPC Number/Migration	(-)	[44]
Health examination	PAI-1/Fibrinogen	(-)	[45,46]
Health examination	Endothelial function	(-)	[52]
Outpatients	PEDF	(-)	[48]
Outpatients	DPP-4	(-)	[49]
Outpatients	Adipose tissue inflammation	(-)	[51]
Outpatients	Vascular inflammation	(-)	[30]
non-DM	Insulin resistance	(-)	[109]
non-DM	LDL-C	(-)	[47]
non-DM	sRAGE	(-)	[58]
non-DM CKD	ADMA	(-)	[57]
T2DM	sRAGE/sVCAM-1	(-)	[53]
T2DM	MCP-1	(-)	[55]
T2DM/non-DM	sRAGE	(-)	[59]
Septic shock patients	IL-6/ADMA	(-)	[56]
JAPAN-ACS sub-study	Plaque progression	(-)	[72]
NASH	HORMA-IR/Adiponectin	(-)	[50]
NBNC-HCC/NASH	NBNC-HCC (↑)	(-)	[88]
EPIC cohort study	Rectal cancer; Colon cancer (No)	(-)	[89]
Schizophrenia	sRAGE	(-)	[99]
Infertile women	Embryonic development /Pregnancy	(-)	[31]
T2DM	TAGE (↓)	α-Glucosidase inhibitor	[64]
T2DM	TAGE (↓), Albuminuria (↓)	DPP-4 inhibitor	[65]
T2DM	TAGE (↓)	Sulfonyl urea	[66]
T1DM/T2DM	TAGE (↓)	Insulin	[54]
T2DM	TAGE (↓)	Statin	[60]
non-DM CKD	TAGE (↓), Proteinuria (↓)	Statin	[61]
Coronary atherosclerosis	TAGE (no effect), sRAGE (↑)	Statin	[62]
SAMIT	TAGE (↓), ANGPTL2 (↓)	Statin	[63]
NASH with dyslipidemia	TAGE (↓)	Statin	[77]
non-DM CRF	TAGE (↓)	Oral adsorbent	[110]
